# A review of piroplasmid infections in wild carnivores worldwide: importance for domestic animal health and wildlife conservation

**DOI:** 10.1186/s13071-016-1808-7

**Published:** 2016-10-10

**Authors:** Mario Alvarado-Rybak, Laia Solano-Gallego, Javier Millán

**Affiliations:** 1 PhD Program in Conservation Medicine, Facultad de Ecología y Recursos Naturales, Universidad Andres Bello, República 252, Santiago, Chile; 2 Centro de Investigación para la Sustentabilidad, Facultad de Ecología y Recursos Naturales, Universidad Andres Bello, República 252, Santiago, Chile; 3 Departament de Medicina i Cirurgia Animals, Facultat de Veterinària, Universitat Autònoma de Barcelona, Bellaterra, Barcelona, Spain; 4 Facultad de Ecología y Recursos Naturales, Universidad Andres Bello, República 252, Santiago, Chile

**Keywords:** Piroplasmids, *Babesia*, *Theileria*, *Cytauxzoon*, *Rangelia*, Wild carnivores, Dog and cat

## Abstract

Piroplasmids are tick-borne protozoan parasites that infect blood cells (erythrocytes, lymphocytes or other leukocytes) or endothelial cells of numerous wild and domestic vertebrates worldwide. They cause severe disease in livestock, dogs, cats, wild mammals and, occasionally, in humans. Piroplasmid infections are prevalent in wild carnivores worldwide although there is limited information about their clinical and epidemiological importance. There are currently nine recognized species of *Babesia*, two of *Theileria*, two of *Cytauxzoon* and one of *Rangelia* infecting captive and wild carnivores, including members of Canidae, Felidae, Mustelidae, Procyonidae, Ursidae, Viverridae, Hyaenidae and Herpestidae in the Americas, Eurasia and Africa. However, the number of piroplasmid species is likely higher than currently accepted due to the reported existence of DNA sequences that may correspond to new species and the lack of studies on many host species and biogeographical areas. Indeed, many species have been recognized in the last few years with the advancement of molecular analyses. Disease and mortality have been documented in some wild carnivores, whereas other species appear to act as natural, subclinical reservoirs. Various factors (e.g. unnatural hosts, stress due to captivity, habitat degradation, climate fluctuation or immunosuppression) have been associated with disease susceptibility to piroplasmid infections in some species in captivity. We aimed to review the current knowledge on the epidemiology of piroplasmid infections in wild carnivores and associated tick vectors. Emphasis is given to the role of wild carnivores as reservoirs of clinical piroplasmosis for domestic dogs and cats, and to the importance of piroplasmids as disease agents for endangered carnivores.

## Background

The incidence and diversity of tick-borne infections in humans and animals have increased in recent years due to several factors. These factors include the existence of better diagnostic tools; increased awareness among the scientific community, veterinarians, physicians and public health authorities; increased contact of humans with wildlife and vectors (urbanization and habitat encroachment); and changes in the environment, such as global climate change [1, 2]. These factors have increased the probabilities of contact with ticks and/or sylvatic reservoir hosts [3].

Piroplasmoses are among the most prevalent arthropod-borne diseases of animals. Piroplasmoses are caused by hemoprotozoan parasites of the phylum Apicomplexa belonging to four related genera: *Babesia*, *Theileria*, *Cytauxzoon* and *Rangelia* [3]. Piroplasmids owe their name to the pear-shaped (pyriform) intracellular stages formed in the host erythrocytes [4]. These parasites have a great economic, veterinary and medical impact worldwide. In fact, they are considered to be the second most commonly found parasites in the blood of mammals after trypanosomes [5], and are frequently found infecting free-living animals worldwide. Thus, they have gained increasing attention as emerging tick-borne diseases [3].

Classification of piroplasmids has largely relied on morphological and biological observations [3, 6]. Formerly, they were classified by: (i) the size and shape of trophozoites in the erythrocytes; (ii) the number of merozoites; and (iii) the host of origin. According to their size, piroplasmids were classified into small and large piroplasmids (mainly in the genus *Babesia*). On the other hand, identification based on host origin was based on the believe that these parasites were strongly host-specific, but this assumption is not longer valid because this is not the case for many species [3, 4, 7, 8]. The sole use of direct observations of blood smears does not allow species identification and molecular techniques are needed [7, 9]. Thus, some of the early descriptions and identifications of piroplamid species were inadequate and did not meet today’s standards. For this reason, only identifications using molecular techniques are reviewed in the present manuscript.

Currently, according to the molecular characterization of multiple gene targets (chiefly 18S rRNA and β-tubulin gene sequences), piroplasmids should be divided into at least five groups: (i) archaeopiroplasmids or Microti group, including small *Babesia* from wild rodents, felids, canids, and other mammals such as hyaenids and procyonids; (ii) prototheilerids or Duncani group, comprising small piroplasmids of cervids, dogs and humans from USA; (iii) babesiids, including primarily canine, bovine, and cervine species; (iv) unguilibabesiids, including primarily bovine, equine, and ovine species; and (v) theileriids, including the genus *Theileria* and *Cytauxzoon* [3, 5, 6, 10]. *Rangelia vitallii* is placed in the clade “*Babesia* (*sensu stricto*)” [11].

In the last few years, there has been a dramatic increase in the number of studies reporting infection with piroplasmids in wildlife. The objective of this paper is to review the current knowledge on the epidemiology of piroplasmid infections in wild carnivore hosts and associated tick vectors. Emphasis is given to the role of wild carnivores as reservoirs of clinical piroplasmosis for domestic dogs and cats, and to the importance of piroplasmids as disease agents for endangered carnivores.

### Natural history of piroplasmids

Although piroplasmoses are among the most relevant diseases of wild and domestic animals [7, 10], many questions remain unsolved concerning their epidemiology and life-cycles. These include questions regarding their phases in the ixodid tick vector as well as the vertebrate host, especially with regard to wildlife [12]. It is known that piroplasmids are maintained in a complex system of vectors and animal reservoirs, and infection of the mammalian host often takes place via the bite of the invertebrate vector, usually ticks [4, 13, 14]. While the tick is feeding, sporozoites are released from its salivary glands and enter the blood stream of the vertebrate host [8, 13]. Parasites then attach to and are endocytosed by erythrocytes (*Babesia* spp.), or initially penetrate into lymphocytes [13] or other leukocytes [15] (*Theileria* spp.), or macrophages, histiocytes, reticuloendothelial cells and/or endothelial cells (*Cytauxzoon* spp. and *R. vitalii*) [16, 17]. This is followed by an intraerythrocytic cycle [4] or intraleukocyte cycle, e.g. in some *Theileria* spp. [18]. Once parasites are in the erythrocytes or leukocytes, they undergo asexual reproduction and merogony, and the daughter cells can infect new cells. A naïve tick then ingests infected erythrocytes. It is unclear whether the transformation from merozoite to gamete (gametocyte) begins in the vertebrate host or in the tick [14]. In the tick midgut, the sexual phase of reproduction occurs when the gametes fuse to form a zygote. The zygote invades the epithelial cell of the tick gut, and an asexual form of reproduction, sporogony, occurs. The resultant forms, ookinetes, leave the epithelial cell and invade either the salivary gland or the ovary of the tick, where they participate in transstadial and transovarial transmissions [4, 5, 8, 11, 14].

Tick bites appear to be the primary manner of transmission for piroplasmids. However, other forms of transmission have been described for some piroplasmid species. For example, direct dog-to-dog transmission for *B. gibsoni* is highly likely and may be the main mode of transmission in some geographical regions such as Australia [19], North America [20–22] and Europe [23, 24]. Vertical transmission is also possible by transplacental infection of pups by *B. gibsoni* [25] and *B. canis* [26] in dogs from Asia. Another route of direct transmission in human babesiosis by *B. microti*, is through blood transfusion in North America [27]. On the other hand, experimental transmission of *Babesia* spp. from domestic to wild animals is usually only successful in closely related species or after splenectomy [8, 28].

### Description of piroplasmid species and prevalence of infection in wild carnivores

**Fig. 1:**
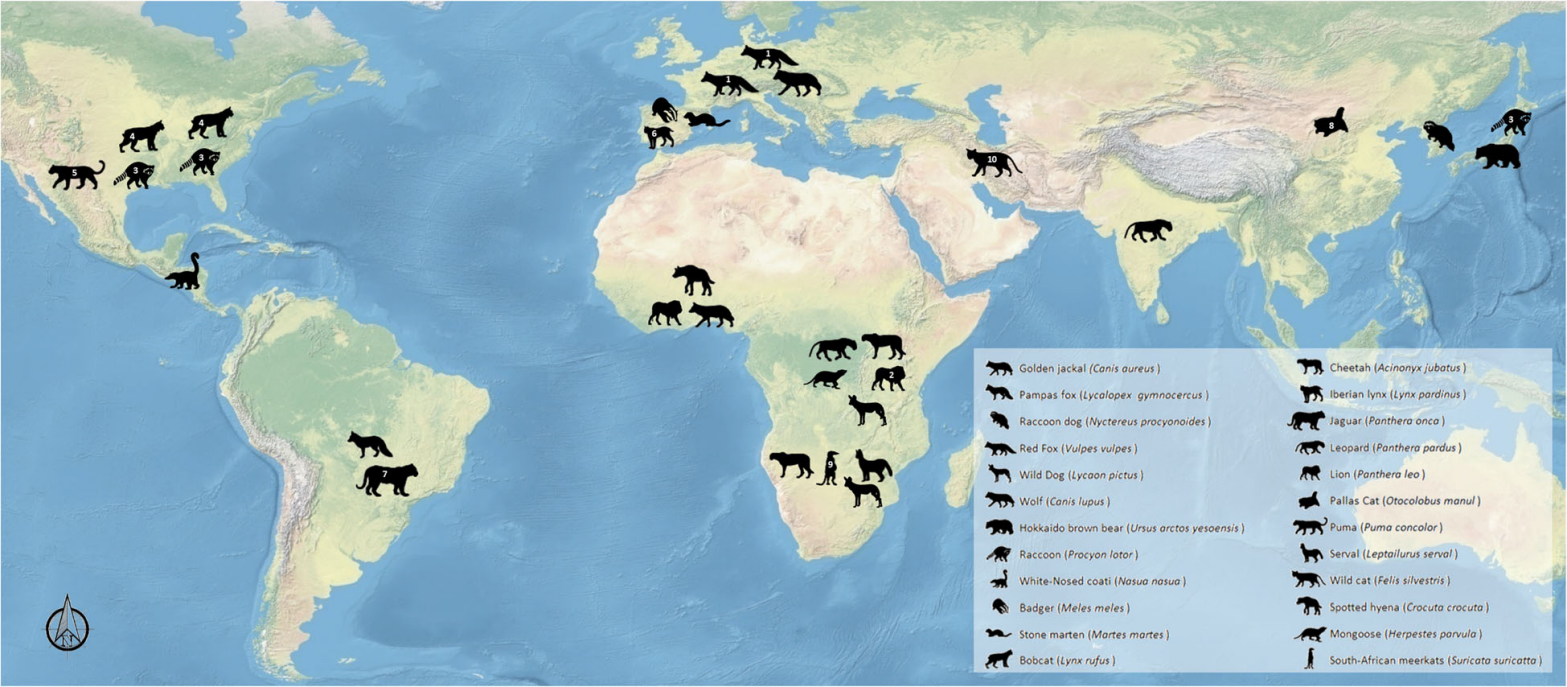
Distribution map of piroplasmid infection in wild carnivores worldwide. (1) High prevalence of *Babesia microti*-like group in red foxes (*Vulpes vulpes*) in Europe suggests that this species may be acting as a sylvatic reservoir for these species, or may even be the natural host of the parasite. (2) A canine distemper epidemic among Serengeti lions (*Panthera leo*) was associated with high levels of *Babesia* during the 1994 and 2001 outbreaks. (3) Raccoons (*Procyon lotor*) in USA and Japan may be uncontrolled reservoirs of *Babesia* sp. and may also participate in the dynamics of human babesiosis caused by *B. microti* as dispersors of infected ticks. (4) Bobcats (*Lynx rufus*) and probably (5) cougars (*Puma concolor*) are the reservoirs of *Cytauxzoon felis* in North America. (6) The Iberian lynx (*Lynx pardinus*) is a natural host for *Cytauxzoon* spp. in the Iberian Peninsula, but due to its reduced population size cannot be considered a relevant reservoir of the parasite. (7) Brazilian wild felids, such as the jaguar (*Panthera onca*), may also be natural hosts for *Cytauxzoon* sp. because infection is never related to the presence of clinical signs. (8) Pallas's cats (*Otocolobus manul*) imported into Oklahoma from Mongolia were found to be infected with intraerythrocytic piroplasms, and DNA sequencing revealed a novel organism, *Cytauxzoon manul*. (9) A meerkat population in South Africa was found to be frequently infected with *Babesia* and *Cytauxzoon* without showing signs of disease. (10) An Asiatic wildcat (*Felis silvestris ornata*) was found suffering from clinical signs of cytauxzoonosis in Iran

In the past few years, important advances have been achieved in the detection and identification of piroplasmids infecting wild carnivores. A wide variety of carnivore species have been reported to be infected with and/or exposed to piroplasmids, including members of the families Canidae, Felidae, Mustelidae, Procyonidae, Ursidae, Viverridae, Hyaenidae and Herpestidae (Fig. 1; Tables 1 and 2).

**Table 1:** Molecular and serological studies performed on *Babesia* spp. and *Theileria* spp. infections in wild carnivores

Host	Reported prevalence		Targeted agent	Country/Region	Sample origin	Observation	Reference
	% (positive/*n*)	Technique					
Canidae							
Bush dog (*Speothos venaticus*)	29.0 (8/27)	IFAT	*Babesia* sp.	Brazil	Z		[66]
Coyote (*Canis latrans*)	0/12	PCR	*B. microti-*like^a^	USA	W		[63]
Crab-eating fox (*Cerdocyon thous*)	5.0 (2/39)	IFAT	*Babesia* sp.	Brazil	Z		[66]
Fennec fox (*Vulpes zerda*)	(1/11)	PCR	*Babesia* sp.	North Africa	W		[126]
Golden jackal (*Canis aureus*)	0/32	PCR	*Babesia* sp.	North Africa	W		[126]
Grey fox (*Urocyon cinereoargenteus*)	26.0 (8/31)	PCR	*B. microti-*like	USA	W		[63]
Hoary fox (*Pseudolopex vetulus*)	0/7	IFAT	*Babesia* sp.	Brazil	Z		[66]
Maned wolf (*Cerdocyon brachyurus*)	0/21	IFAT	*Babesia* sp.	Brazil	Z		[66]
Pale fox (*Vulpes pallida*)	4.0 (1/28)	PCR	*Babesia* sp.	North Africa	W		[126]
Racoon dog (*Nyctereutes procyonoides*)	(3/14)	PCR	*B. microti-*like	South Korea	W	Emaciated. One with severe tick infestation	[62]
Red fox (*Vulpes vulpes*)	1.1 (1/91)	PCR	*B. canis*	Portugal	W		[53]
	0.7 (1/138)	PCR	*Babesia* sp.	Northeastern Poland	W		[63]
	0/13	PCR	*B. microti-*like	Sicily, Italy	W		[127]
	0/16	PCR	*Babesia* sp.	North Africa	W		[126]
	0.8 (1/119)	PCR	*B. canis*	Bosnia	W		[58]
	50.0 (10/20)	PCR	*B. microti-*like	Spain	W		[128, 129]
	(1/2)	PCR	*B. microti-*like	Italy	W		[130]
	(1/5)	PCR	*B. microti-*like	Spain	W		[55]
	37.0 (58/158)	PCR	*B. microti-*like	USA	W		[63]
	(1/1)	PCR	*B. microti-*like	Prince Edward Island, Canada	W	Weakness, anemia, non-suppurative meningoencephalitis, bronchopneumonia and vacuolar hepatopathy	[64]
	5.2 (10/191)	PCR	*B. microti-*like	Croatia	W		[59]
	0.5 (1/191)	PCR	*B. microti-*like	Croatia	W		[59]
	69.2 (63/91)	PCR	*B. microti-*like	Portugal	W		[53]
	50.0 (18/36)	PCR	*B. microti-*like	Austria	W		[56]
	46.0 (121/261)	PCR	*B. microti-*like	Thuringia, Germany	W	Carcasses with high infestations of ticks	[60]
	0.98 (2/205)	PCR	*B. microti-*like	Italy	W		[12]
	20.0 (81/404)	PCR	*B. microti-*like	Hungary	W		[57]
	31.9 (38/119)	PCR	*B. microti-*like	Bosnia	W	9.2 % co-infection with *Hepatozoon canis;* one fox co-infected with *B. canis* and *H. canis*	[58]
	(2/12)	PCR	*B. microti-*like	Catalonia, Spain	W	One fox co-infected with *Coxiella burnetti*	[83]
	14.6 (46/360)	PCR	*B. microti-*like		W/R		[131]
Ruppell fox (*Vulpes rueppellii*)	0/11	PCR	*Babesia* sp.	North Africa	W		[126]
Side-striped jackal (*Canis adustos*)	0/2	PCR	*Babesia* sp.	North Africa	W		[126]
Wild dog (*Lycaon pictus*)	5.3 (16/301)	PCR	*B. rossi*	South Africa	W		[132]
	0/11	PCR	*Babesia* sp.	Zambia	W	Co-infection with *Hepatozoon* sp.	[109]
Wolf (*Canis lupus*)	0/7	PCR	*B. microti-*like	Italy	W		[12]
	0/3	IFAT	*Babesia* sp.	Brazil	Z		[66]
	(2/12)	PCR	*B. canis*	Budapest, Hungary	R	Good body condition. Necropsy with severe jaudice, liver, gall bladder and spleen enlarged	[106]
	0/7	PCR	*Babesia* sp.	Italy	W		[12]
Ursidae							
Hokkaido brown bear (*Ursus arctos yesoensis*)	(1/1)	PCR	*Babesia* sp. UR1	Hokkaido, Japan	W	Heavily infested with ticks and anemia. Co-infection with *Cytauxzoon* sp.	[79]
Japanese black bear (*Ursus thibetanus japonicus*)	14.1 (22/156)	PCR	*Babesia* sp.	Iwate, Japan	W	76.3 % co-infection with *Hepatozoon ursi*	[48]
Procyonidae							
Raccoon (*Procyon lotor*)	8.3 (2/24)	PCR	*Babesia* sp.	Hokkaido, Japan	W	All have splenomegaly	[44]
	(1/1)	PCR	*Babesia* sp.	Illinois, USA	W	Anemia	[46]
	90.0 (37/41)	PCR	*Babesia* sp.	North Carolina, USA	W	67 % co-infection with *B. microti-*like	[89]
	83.0 (34/41)	PCR	*B. microti-*like	North Carolina, USA	W	76 % co-infection with *Babesia* (*sensu stricto*) clade	[89]
	1.7 (6/348)	PCR	*Babesia* sp.	Hokkaido, Japan	W	Heavily infested with ticks	[47]
	(14/17)	PCR	*B. microti-*like	Florida, USA	W		[45]
White-nosed coatis (*Nasua narica*)	100 (20)	PCR	*Babesia* sp.	Costa Rica	W		[133]
Mustelidae							
American mink (*Neovison vison*)	(13/13)	PCR	*Babesia* sp. NV-1	Hokkaido, Japan	W		[134]
Badger (*Meles meles*)	(1/5)	PCR	*Babesia* sp.	Burgos, Spain	W		[55]
North American river otter (*Lontra canadensis*)	82.0 (32/39)	PCR	*Babesia* sp.	North Carolina, USA	W	Wild-caught	[135]
Stone marten (*Martes foina*)	(1/10)	PCR	*B. vogeli*	Catalonia, Spain	W	Co-infection with *Bartonella clarridgeiae*	[83]
Felidae							
Black-footed cat (*Felis nigripes*)	(5/8)	PCR	*Babesia* sp.	Swaziland, Southern Africa	R		[65]
Caracal (*Caracal caracal*)	(1/1)	PCR	*Babesia* sp.	Durban, South Africa	W	Found ill with clinical sign of feline babesiosis	[136]
	0/2	PCR	*Babesia* sp.	Swaziland, Southern Africa	W		[65]
	0/1	IFAT	*Babesia* sp.	Brazil	Z		[66]
Cheetah (*Acinonyx jubatus*)	19.0 (18/97)	PCR	*B. felis*	Namibia	R		[65]
	6.1 (3/49)	PCR	*B. felis*	Namibia	W		[65]
	3.0 (3/97)	PCR	*B. leo*	Namibia	R		[65]
	28.5 (39/137)	D/PCR	*B. lengau*	South Africa	R		[137]
	(5/5)	PCR	*Theileria* sp.	Salama-Malili ranch, Kenia	R	Subclinical	[69]
	0/5	PCR	*Babesia* sp.	Salama-Malili ranch, Kenia	R	Subclinical	[69]
	(4/4)	PCR	*B. leo*	Zimbabwe	R		[65]
Fishing cat (*Prionailurus viverrinus*)	0/1	IFAT	*Babesia* sp.	Brazil	Z		[66]
Jaguar (*Panthera onca*)	(6/13)	IFAT	*Babesia* sp.	Brazil	Z		[66]
Leopard (*Panthera pardus*)	(1/1)	PCR	*B. leo*	Namibia, Swaziland, South Africa	R		[65]
	(2/2)	PCR	*Babesia* sp.	Nairobi National Park, Kenya	R	Subclinical	[69]
	0/1	IFAT	*Babesia* sp.	Brazil	Z		[66]
Lion (*Panthera leo*)	(16/16)	D/PCR	*Babesia* sp.	Kruger National Park, South Africa	W	Blood samples	[136]
	12.0 (3/25)	PCR	*B. leo*	Swaziland, Southern Africa	R	Co-infection with *B. felis*	[65]
	25.0 (14/56)	PCR	*B. leo*	Swaziland, Southern Africa	W	Co-infection with *B. felis*	[65]
	12.0 (3/25)	PCR	*B. felis*	Swaziland, Southern Africa	R		[65]
	1.7 (1/56)	PCR	*B. felis*	Swaziland, Southern Africa	W		[65]
	89.5 (238/266)	PCR	*Babesia* sp.	Serengeti	W	1994 Canine distemper virus epidemic	[93], Terio personal communication
	97.0 (34/35)	PCR	*Babesia* sp.	Ngorongoro	W	2001 Canine distemper virus epidemic	[93], Terio personal communication
	0/12	IFAT	*Babesia* sp.	Brazil	Z		[66]
	(2/2)	D/PCR	*B. canis*	Nairobi Orphanage, Kenya	R	Anemia, lethargy, wobble movement and dry eyes	[69]
	12.0 (10/86)	PCR	*B. vogeli*	Zimbabwe	R		[67]
	59.0 (51/86)	PCR	*B. leo*	Zimbabwe	R		[67]
	25.0 (6/24)	PCR	*B. felis*	Zambia	W	Co-infection with *Hepatozoon* sp.	[109]
	25.0 (6/24)	PCR	*B. leo*	Zambia	W	Co-infection with *Hepatozoon* sp.	[109]
	1.0 (1/86)	PCR	*T. sinensis*	Zimbabwe	R		[67]
	1.0 (1/86)	PCR	*T. parva*	Zimbabwe	R		[67]
Little spotted cat (*Leopardus tigrinus*)	24.0 (9/38)	IFAT	*Babesia* sp.	Brazil	Z		[66]
Margay (*Leopardus wiedii*)	(2/4)	IFAT	*Babesia* sp.	Brazil	Z		[66]
Ocelot (*Leopardus pardalis*)	60.0 (26/43)	IFAT	*Babesia* sp.	Brazil	Z		[66]
Pampas cat (*Oncifelis colocolo*)	(3/5)	IFAT/PCR	*Babesia* sp.	Brazil	Z		[66]
Puma (*Puma concolor*)	78.0 (32/41)	PCR	*Babesia* sp.	Florida, USA	W	5 % co-infection with *C. felis*	[119]
	(2/18)	IFAT	*Babesia* sp.	Brazil	Z		[66]
Tiger (*Panthera tigris*)	0/6	IFAT	*Babesia* sp.	Brazil	Z		[66]
Serval (*Leptailurus serval*)	(1/3)	PCR	*B. felis*	Swaziland, Southern Africa	R		[65]
	(2/2)	PCR	*B. vogeli*	Zimbabwe	R		[67]
	0/1	IFAT	*Babesia* sp.	Brazil	Z		[66]
Wild cat (*Felis silvestris*)	(6/6)	PCR	*B. vogeli*	Zimbabwe	R		[67]
Yaguarundi (*Puma yagouaroundi*)	25.0 (6/25)	IFAT	*Babesia* sp.	Brazil	Z		[66]
Viverridae							
Common genet (*Genetta genetta*)	(1/2)	IFAT/PCR	*Babesia* sp.	Brazil	Z		[66]
	0/34	PCR	*Babesia* sp.	Catalonia, Spain	W		[83]
Hyaenidae							
Spotted hyena (C*rocuta crocuta*)	(6/19)	PCR	*Babesia* sp.	Zambia	W	Co-infection with *Hepatozoon* sp.	[109]
Herpestidae							
South-African meerkats (*Suricata suricatta*)	80.0 (37/46)	D/PCR	*Babesia* sp.	Kalahari, South Africa	W	46 % of co-infection with *Cytauxzoon* sp.	[78]

^a^We used the name *Babesia microti*-like for all isolates belonging to the *B. microti* group and reported by their authors as *B. microti*-like, "*T. annae*", "*B. annae*" or "*B. vulpes*"

*Abbreviations*: *D* direct examination of smear, *H* histology, *IFAT* Indirect Fluorescent Antibody Test, *PCR* Polymerase Chain Reaction, *Z* Zoo collection, *R* rehabilitation center, *W* wild animal

**Table 2:** Molecular studies of *Rangelia vitalii* and *Cytauxzoon* sp. infections in wild carnivores

Host	Reported prevalence		Targeted agent	Country/Region	Sample origin	Observation	Reference
	% (positive/*n*)	Technique					
Canidae							
Crab-eating fox (*Cerdocyon thous*)	30.0 (6/20)	PCR	*R. vitalii*	Brazil	W		[11]
	(1/1)	PCR	*R. vitalii*	Brazil	W	Cachexia and intense dehydration, conjunctiva and oral mucosae were distinctly pale. Co-infection with canine distemper virus.	[73]
Pampas fox (*Lycalopex gymnocercus*)	0/4	PCR	*R. vitalii*	Brazil	W		[11]
	(1/1)	PCR	*R. vitalii*	Brazil	W	Mucosae were moderately pale. Spleen with moderate follicle hyperplasia and extramedullary hematopoiesis.	[73]
	(1/1)	PCR	*R. vitalii*	Brazil	W	Kidney with hyaline degeneration and coagulation necrosis. Liver with slight vacuolar degeneration. Spleen with red pulp hyperplasia.	[72]
Ursidae							
Hokkaido brown bear (*Ursus arctos yesoensis*)	(1/1)	PCR	*Cytauxzoon* sp.	Hokkaido, Japan	W	Heavily infested with ticks and anemia. Co-infection with *Babesia* sp. UR1	[79]
Felidae							
Bobcat (*Lynx rufus*)	33.0 (10/30)	PCR	*C. felis*	North Carolina, USA	W	Region where cytauxzoonosis is prevalent in domestic cat	[138]
	7.0 (5/69)	PCR	*C. felis*	Pennsylvania, USA	W	Region where cytauxzoonosis is not prevalent in domestic cat	[138]
	25.6 (34/133)	PCR	*C. felis*	Arkansas, USA	W		[105]
	20.0 (138/696)	PCR	*C. felis*	13 states, USA	W		[90]
	0/1	PCR	*C. felis*	USA	R		[139]
Wild cat (*Felis silvestris ornata*)	(1/1)	D/PCR	*C. felis*	Iran	W	Cachexia and anemia	[140]
European wildcat (*Felis silvestris silvestris*)	14.3 (3/21)	PCR	*Cytauxzoon* sp.	Italy	W	Road kill animals	[121]
Iberian lynx (*Lynx pardinus*)	1.9 (1/51)	D/PCR	*Cytauxzoon* sp.	Sierra Morena, Spain	W	One injured young male	[76]
	15.0 (3/20)	PCR	*Cytauxzoon* sp.	Sierra Morena, Spain	W		[75]
	75.0 (24/32)	PCR	*C. felis*	Sierra Morena, Spain	W		[77]
	0/45	PCR	*C. felis*	Doñana, Spain	W		[77]
	26.9 (25/112)	PCR	*C. felis*	Sierra Morena, Spain	W/R	22 % co-infection with *Toxoplasma gondii*	[141]
Jaguar (*Panthera onca*)	(1/6)	PCR	*Cytauxzoon* sp.	Brazil	R		[111]
	(26/26)	PCR	*C. felis*	Pantanal, Brazil	W		[142]
Little spotted cat (*Leopardus tigrinus*)	0/14	PCR	*Cytauxzoon* sp.	Brazil	R		[111]
Lion (*Panthera leo*)	0/266	PCR	*C. felis*	Serengeti	W	1994 Canine distemper virus epidemic	[93]
	0/35	PCR	*C. felis*	Ngorongoro	W	2001 Canine distemper virus epidemic	[93]
	0/1	PCR	*C. felis*	USA	R		[139]
	2.0 (2/86)	PCR	*C. manul*	Zimbabwe	R		[67]
Margay (*Leopardus wiedii*)	0/2	PCR	*Cytauxzoon* sp.	Brazil	R		[111]
Ocelot (*Leopardus pardalis*)	6.8 (2/29)	PCR	*Cytauxzoon* sp.	Brazil	R		[111]
Pallas's cat (*Otocolobus manul*)	(4/4)	H/D/PCR	*C. manul*	Mongolia	W	Trapped in Mongolia and transported to USA	[74, 122, 143]
	0/3	PCR	*Cytauxzoon* sp.	Brazil	R		[111]
Puma (*Puma concolor*)	12.0 (5/41)	PCR	*C. felis*	Florida, USA	W	5 % co-infection with *Babesia* sp.	[119]
	(3/3)	D/PCR	*C. felis*	Florida, USA	R	1 cougar with anorexia and lethargy. Hematological findings of mild hemolytic anemia	[113]
	(2/9)	PCR	*Cytauxzoon* sp.	Brazil	R		[111]
	(1/7)	PCR	*C. felis*	USA	R		[90]
	0/1	PCR	*C. felis*	USA	R		[139]
Serval (*Leptailurus serva*l)	0/1	PCR	*C. felis*	USA	R		[90]
Tiger (*Panthera tigris*)	(4/8)	PCR	*C. felis*	USA	Z		[139]
Yaguarundi (*Puma yagouaroundi*)	0/6	PCR	*Cytauxzoon* sp.	Brazil	R		[111]
Viverridae							
Common genet (*Genetta genetta*)	0/10	PCR	*Cytauxzoon* sp.	Doñana, Spain	W		[144]
Herpestidae							
Egyptian mongoose (*Herpestes ichneumon*)	0/24	PCR	*Cytauxzoon* sp.	Doñana, Spain	W		[144]
South African meerkats (*Suricata suricatta*)	57.0 (26/46)	D/PCR	*Cytauxzoon* sp.	Kalahari, South Africa	W	46 % of co-infection with *Babesia* sp.	[78]

*Abbreviations*: *D* direct examination of smear, *H* histology, *IFAT* Indirect Fluorescent Antibody Test, *PCR* Polymerase Chain Reaction, *Z* Zoo collection, *R* rehabilitation center, *W* wild animal

Historically, the presence of piroplasmid species in wild carnivores was believed to be an incidental finding unrelated to disease and was described under other generic names, e.g. *Piroplasma*, *Nuttalia* and *Nicollia*, to name a few. This was due to the fact that the diagnosis was based solely on morphology [8, 29]. The first piroplasmid reported in a wild canivore received the name of *Babesia herpestidis* because it was observed in a blood smear of an Egyptian mongoose (*Herpestes ichneumon*) caught in Lisbon in 1908 [30]. Intracellular pyriform structures in the erythrocytes, 1.5 to 1.8 μm in length, which differed from those described thus far in horses and deers, were noted [30]. However, as mentioned previously, species descriptions of older findings based on morphology alone are most likely unreliable. Currently, with a lack of reference material, it is almost impossible to identify many of these piroplasmids with some degree of accuracy. Therefore, in the present review, we relied only on molecular identification of piroplasmid species classified as *Babesia* spp., *Theileria* spp., *Rangelia* spp., and *Cytauxzoon* spp., without taking into account descriptions made on the basis of morphology alone. With this criterion, there are currently nine recognized species of *Babesia*, two of *Theileria,* two of *Cytauxzoon,* and one of *Rangelia* infecting wild carnivores worldwide.

#### *Babesia* spp.

Infection by *Babesia* spp. has been reported in 33 carnivore species belonging to eight families in Europe, Africa, America and Asia (Table 1). Serological evidence of exposure has been reported in ten additional species. Taking into account studies with representative sample sizes, reported molecular prevalences of *Babesia* spp. infection vary widely between 0.5–100 % depending on the species and location (Table 1). *Babesia* spp. infections by means of direct diagnosis techniques such as blood smear examination have been described for several carnivore species [30–41]. These descriptions of *Babesia* species are insufficient and do not meet today’s accepted standards. Then, taking into account molecular diagnosis, infections with nine species of *Babesia* have been reported thus far: *B. canis*, *B. rossi* and *B. vogeli*, most commonly in canids; *B. leo*, *B. felis* and *B. lengau* in felids; the piroplasmids belonging to the *B. microti-*like group ("*Theileria annae*"; “Spanish dog isolate”; *Babesia* cf. *microti*; "*Babesia vulpes*") that commonly infect some species of wild canids; and two potentially new species, named *Babesia* NV-1 in the American mink *Neovison vison* and *Babesia* UR1 in the Hokkaido brown bear (*Ursurs arctos yesoensis*), both in Japan (Table 1). In addition to those, it has been proposed that more than one species of *Babesia* may parasitize the raccoon (*Procyon lotor*). Before the molecular era, this agent was named *Babesia lotori* [42]. However, in the last years, molecular analyses carried out in North America and Japan (where this species was introduced) identified several sequences corresponding with two or more species of *Babesia*. One of these sequences, first detected by Goethert & Telford [43] in Massachusetts, USA, and later by Kawabuchi et al. [44] in Japan and Clark et al. [45] in Florida, USA, is phylogenetically related with the "Spanish dog isolate" (*B. microti-*like group) and piroplasmid sequences obtained from skunks and red foxes. A second potential species was detected by Birkenheuer et al. [46] in Illinois. The obtained complete sequence of the 18S rRNA gene was most closely related with a sequence obtained from an *Ixodes ovatus* tick infesting a dog in Japan. This agent was subsequently detected in Japanese raccoons by Jinnai et al. [47], confirming that both species are also present in Japan. Interestingly, a sequence showing 99.3 % identity with these agents was later detected in a Japanese black bear (*Ursus thibetanus japonicus*) [48]. Moreover, other *Babesia* spp. sequences identified by Jinnai et al. [47] were further separated into a novel phylogenetic group, indicating that at least three species of *Babesia* may infect feral raccoons in Japan.

Regarding "*T. annae*"[49], Baneth et al. [50] recently reclassified this piroplasm as a new species named "*B. vulpes*". However, although there is a consensus about this agent being a *Babesia* and not a *Theileria* [50], both names are *nomina nuda* and thus unavailable (see Harris [51]). In this review, we will use the name "*Babesia microti*-like group" as recommended by Harris [51]. Isolates of *Babesia microti*-like group cause clinical disease in dogs, and the most likely natural reservoir is the red fox *Vulpes vulpes* [24, 49, 52]. The geographical distribution of infected red foxes includes southern Europe (Portugal [53], Spain [54, 55], Italy [12], Austria [56], Hungary [57], Bosnia [58], Croatia [59], Germany [60] and Poland [61]); South Korea in Asia [62]; and North America [63, 64]. Observed prevalences in the red fox range from 5 % in Croatia [59] to 69 % in Portugal [53]. In other carnivore species not belonging to the family Canidae, *B. microti*-like group has been reported with high prevalences (up to 83 %) in the USA (Table 1). A *Babesia microti-*like group agent was also detected in raccoon dogs (*Nyctereutes procyonoides*) from South Korea [62]. Alhough Baneth et al. [49] classified this agent as a “*Babesia* sp. 2 raccoon”, and discussed the phylogeny of the parasite as if the raccoon and the raccoon dog were the same species, it is worth noting that the raccoon dog belongs to the family Canidae and not to the family Procyonidae (as the raccoon does). Therefore, this parasite is most likely a "*B. annae*" isolate as other *B. microti*-like group agents parasitizing canids worldwide.

Interestingly, studies in free-ranging lions indicate that co-infections with different species of piroplasmids (*B. leo* and *B. felis*) were common in South Africa [65]. Table 1 summarizes other studies were co-infections with more than one piroplasmid species in wild carnivores have been found.

Reports from serological surveys of piroplasmids are scarce and information is available only for *Babesia* species in Brazilian wild carnivores (Table 1). André et al. [66] reported seroprevalences of 5 % in the crab-eating fox (*Cerdocyon thous*), 11 % in puma (*Puma concolor*), 24 % in little spotted cat (*Leopardus tigrinus*), 29 % in bush dog (*Speothos venaticus*), 25 % in yaguarundi (*Puma yagouaroundi*), 46 % in jaguar (*Panthera onca*), 50 % in margay (*Leopardus wiedii*), 60 % in pampas cat (*Oncifelis colocolo*), and 60 % in ocelots (*Leopardus pardalis*).

#### *Theileria* spp.

Only two species of *Theileria* have been described in free-living carnivores, namely *T. sinensis* and *T. parva* (Table 1), both found infecting captive lions [67]. *Theileria parva* is the agent of the Corridor Disease and East Coast Fever in cattle and African buffalo [15], and *T. sinensis* was reported to infect cattle and yaks in China [68]. Neither of these species was described previously in felids and further genomic studies are needed to characterize these organisms in felids [67]. Interestingly, Githaka et al. [69] inferred from phylogenetic analyses that a piroplasmid detected in cheetahs in Kenya was closely related to a *Theileria* sp. that infects sheep and giraffes. In summary, these cases of carnivores infected by piroplasmids of herbivores are probably the result of spill-overs from the latter and may have little relevance at the population level.

#### *Rangelia* spp*.*

Only one species, *Rangelia vitalii*, has been described (Table 2). This piroplasmid causes the canine rangeliosis, a severe tick-borne hemorrhagic disease of domestic dogs in Brazil, Argentina and Uruguay [70, 71]. *Rangelia vitalii* infection has been described only in two species of wild canids, the crab-eating fox (*Cerdocyon thous*), with a prevalence of infection of 30 % [11], and the pampas fox (*Lycalopex gymnocercus*), with two individual cases in Brazil [72, 73].

#### *Cytauxzoon* spp.

Infections with *Cytauxzoon* spp. have been reported almost exclusively in felids (Table 2). There is currently only one unquestioned accepted species of *Cytauxzoon*, namely *C. felis*, which infects North American felids [bobcats (*Lynx rufus*) and pumas (*Puma concolor*)] (Table 2). Another species, *C. manul*, was described based on material from the Pallas’s cat (*Otocolobus manul*) from Mongolia, and the percent sequence divergence between this parasite and *C. felis* allowed the authors to consider this as a distinct species [74]. However, many questions remain regarding *Cytauxzoon* taxonomy. For example, the identification of *C. felis* as the causative agent of infection outside America is probably incorrect. In this regard, the sequencing of a 1,726-bp region of the 18S rRNA gene of piroplasmids in the Iberian lynx (*Lynx pardinus*) supported the distinction between American and Eurasian *Cytauxzoon* spp. and suggested that different species or strains may exist in different geographical locations [75]. Surprisingly, three *Cytauxzoon* sequences from Iberian lynx were more closely related to the sequence obtained from a Spanish cat than to a fourth sequence from another Iberian lynx, which clustered together with *C. manul* [75]. This indicates that *Cytauxzoon* taxonomy remains far from resolved.

Observed prevalences of infection by *Cytauxzoon* vary between species and locations. In the bobcat, the species for which most information was gathered, the prevalence varies from 7 % in low-endemic areas to 33 % in endemic regions of the USA (Table 2); similar prevalences have been reported in pumas living in the same regions. In the Iberian lynx, the parasite is apparently present only in one of its two main metapopulations (namely at Doñana and Sierra Morena), as infection has never been demonstrated in any of the lynx analyzed from Doñana [75–77]. In Sierra Morena, observed prevalences ranged between 15 and 75 % depending on the study (Table 2).

The only species reported to be infected by *Cytauxzoon* not belonging to the family Felidae is the South African meerkat (*Suricata suricatta*; family Herpestidae) and the Hokkaido brown bear (family Ursidae). In the case of the meerkat, a single study reported a prevalence of 57 % in 46 animals sampled in the Kalahari [78]; this species lives on ranchlands in close proximity to human settlements, which may have increased the potential for pathogen interspecific transmission [78]. In the case of the Hokkaido brown bear, only a single case was reported [79].

### Tick vectors of infection

As mentioned above, piroplasmoses are generally tick-borne diseases. However, few studies have attempted to determine the tick species transmitting piroplasmids in the wild, and only few have determined the presence of piroplasmids in ticks retrieved from wild carnivores (Table 3).

**Table 3:** Reported body of evidence of *Babesia* spp. and *Cytauxzoon* spp. isolates in ticks found on wild carnivores

Host from which ticks were retrieved	Reported prevalences		Targeted agent	Tick species	Region/​Country	Reference
	% (positive/*n*)	Technique				
Canidae						
Red fox (*Vulpes vulpes*)	1/3 adults	PCR	*B. microti-*like^a^	*Ixodes hexagonus*	Burgos, Spain	[81]
	0/2 nymphs	PCR	*B. microti-*like	*Ixodes hexagonus*	Burgos, Spain	[81]
	(8/870)	PCR	*B. microti-*like	*Ixodes ricinus*	Thuringia, Germany	[60]
	(19/585)	PCR	*B. microti-*like	*Ixodes canisuga*	Thuringia, Germany	[60]
	(13/485)	PCR	*B. microti-*like	*Ixodes hexagonus*	Thuringia, Germany	[60]
	(4/870)	PCR	*B. microti* isolate	*Ixodes ricinus*	Thuringia, Germany	[60]
	(1/585)	PCR	*B. microti* isolate	*Ixodes canisuga*	Thuringia, Germany	[60]
	(1/485)	PCR	*B. microti* isolate	*Ixodes hexagonus*	Thuringia, Germany	[60]
	1 pool of 20 ticks	PCR	*B. microti-*like and *B. vogeli*	*Rhipicephalus turanicus*	Catalonia, Spain	[83]
Ursidae						
Hokkaido brown bear (*Ursus arctos yesoensis*)	0/1	PCR	*Cytauxzoon* sp.	*Ixodes ovatus*	Hokkaido, Japan	[79]
Mephitidae						
Striped skunk (*Mephitis mephitis*)	19.4 (6/31)	PCR	*B. microti-*like	*Ixodes scapularis*	New York, USA	[82]
Procyonidae						
Raccoon (*Procyon lotor*)	23.5 (93/396)	PCR	*B. microti-*like	*Ixodes scapularis*	New York, USA	[82]
Felidae						
Bobcat (*Lynx rufus*)	na	D	*Cytauxzoon* sp.	*Dermacentor variabilis*	Oklahoma, USA	[86]
Lion (*Pathera leo*)	na	PCR	*Babesia* sp.	*Stomoxys* sp.	Ngorongoro	[93]
Mustelidae						
Stone marten (*Martes foina*)	1 pool of 12 ticks	PCR	*B. vogeli*	*Rhipicephalus turanicus*	Catalonia, Spain	[83]
Eurasian badger (*Meles meles*)	1 pool of 18 nymphs	PCR	*B. microti-*like	*Ixodes canisuga*	Catalonia, Spain	[83]
Viverridae						
Common genet (*Genetta genetta*)	1 pool of 3 ticks	PCR	*Babesia* sp.	*Ixodes ventalloi*	Catalonia, Spain	[83]

^a^We used the name *Babesia microti*-like for all isolates belonging to the *B. microti* group and reported by their authors as *B. microti*-like, "*T. annae*", "*B. annae*" or "*B. vulpes*"

*Abbreviations*: *D* direct examination of smear, *PCR* Polymerase Chain Reaction

In dogs, *Rhipicephalus sanguineous, Dermacentor reticulatus* and *Haemaphysalis elliptica* (formerly *Haemaphysalis leachi*) are the recognized vectors of *B. vogeli*, *B. canis* and *B. rossi*, respectively [23]. In cats, the vectors of babesiosis are unknown [14]. In wildlife, *Ixodes hexagonus* was considered the leading candidate as a vector responsible for the infection of domestic dogs with *B. microti*-like group, but solely based on an association between the presence of this tick species on dogs at the time they were diagnosed [63–80]. In agreement with this, *B. microti*-like group isolate was detected (as "*Theileria annae*") in one of three adult *Ixodes hexagonus* infesting foxes in Spain [81].

In a larger survey carried out in Thuringia, Germany, Najm et al. [60] detected *B. microti*-like group in *Ixodes ricinus*, *Ixodes canisuga* and *I. hexagonus*, also retrieved from foxes. This study also detected isolates of *B. microti*-like group (two different genotypes) in the same species of tick, but this probably reflects that these ticks became infected after feeding on micromammals and not foxes. This may also be the case of the *B. microti*-positive ticks retrieved from striped skunk (*Mephitis mephitis*) and raccoons (*Procyon lotor*) in New York, USA [82]. In Spain, a recent study revealed the presence of *B. microti*-like group in a pool of nymphs of *I. canisuga* from a badger (*Meles meles*), but the badger was not infected [83]. Also in that study, a pool of *Rhipichephalus turanicus* from a red fox was co-infected with *B. microti*-like group and *B. vogeli*, but in that case, the host was indeed found to be infected by *B. microti*-like group. A further pool of *Rh. turanicus* from an uninfected stone marten (*Martes foina*) was also infected with *B. vogeli* [83]. Though much stronger evidence is necessary to probe this hypothesis, *Rhipicephalus* ticks might have a role as vectors of *Babesia* spp. other than *B. vogeli*. Finally, Shock et al. [84] identified DNA of a *Babesia* similar to *Babesia poelea*-like species in a *Dermacentor variabilis* pool from a raccoon in the USA. However, all these tick/parasite associations do not imply effective transmission of the parasite by the tick species.

The life-cycle of *C. felis* in North America is the best known cycle among the piroplasmids of carnivores. The parasite has been recovered from two tick species, *D. variabilis* and *Amblyomma americanum*, but competence has only been demonstrated in the latter [85]. In fact, the geographic range of the parasite overlaps with the ranges inhabited by *A. americanum* and the bobcat [85]. *Dermacentor variabilis* was experimentally demonstrated to transmit *C. felis* from wild felids to domestic cats according to one study [86], but this was not confirmed in a later investigation [87]. The vector for other *Cytauxzoon* sp. in other locations is not known. In Brazil, *Amblyomma cajennense* or another ixodic tick has been proposed as a vector, because this tick was found in a captive-reared lion with fatal cytauxzoonosis [88]. In the Iberian Peninsula, no attempt has been made to determine the identity of the tick vector. The potential absence of the tick vector may be the cause of the absence of *Cytauxzoon* sp. in the lynx population in Doñana [75]. Finally, *Cytauxzoon* sp. DNA was detected in one *Ixodes ovatus* from a Japanese brown bear suffering from cytauxzoonosis [79].

It is worth noting that in populations of wild carnivores with high prevalences of piroplasmid infections, it may be possible for the parasite to be maintained in the vertebrate host without the participation of tick vectors through transplacental [25, 89] or direct transmission by bites [20]. This may explain the maintenance of infection in some species of wild carnivores in different geographical regions that may not have competent tick vectors.

### Pathological, population effects and potential impact of piroplasmoses on wild carnivore conservation

Piroplasmid infections in wild animals are typically subclinical [8, 49, 90, 91]. For example, there is some evidence that indigenous African canids can harbour *B. rossi* without showing clinical signs of disease, contrary to what happens in dogs, suggesting that wild canids in Africa have been historically exposed to this piroplasmid [92]. Nevetheless, piroplasmids can be pathogenic under certain circumstances such as when they parasitize an unnatural host, the host is stressed due to captivity or is immunosupressed, or there is habitat degradation or climate fluctuations [8, 93, 94]. Moreover, piroplasmids can occasionally cause severe disease in domestic animals (e.g. [3, 95, 96]), humans [3, 4, 97] and also wild mammals [8, 64]. The clinicopathological abnormalities of piroplasmoses in domestic and wild ruminants are usually fever, anemia and hemoglobinuria [8, 98]. Piroplasmids can also affect marsupials belonging to the family Macropodidae with anemia, lethargy and inappetence [99]. Due to the scarcity of studies about the pathology and clinical features of piroplasmosis in wild carnivores, inferences about the potential pathological effects must be made based on data from their domestic counterparts. For example, most cats affected by feline babesiosis caused by *B. felis* are adults of less than three years of age and present with clinical signs such as anorexia, listlessness, and anemia, followed by icterus, with an estimated mortality of about 15 % [100]. Intriguingly, *B. felis* infection is not associated with fever [100, 101]. On the other hand, most common clinical signs and clinicopathological abnormalities in domestic dogs infected with *B. gibsoni* include anorexia, lethargy, vomiting, fever, anemia and hemoglobinuria [14, 51]. Infection by *B. microti*-like group in dogs causes mainly hemolytic regenerative anemia, thrombocytopenia, pale mucous membranes, anorexia and apathy [102]. Some studies have reported high fatality rates (22 %) [103, 104]. Cytauxzoonosis due to *C. felis* in domestic cats is typically acute and fatal, and is characterized by fever, anorexia, listlessness, anemia, icterus and usually death within 19–21 days [105]. However, recent evidence indicates that cat survival of *C. felis* infection is higher than previously believed and subclinical infections have been identified [85].

#### *Babesia* spp.

*Babesia* spp. infections normally occur as clinically unapparent infections in immunocompetent hosts [8, 93]. Mortalities have rarely been reported in free-ranging and captive carnivores. When mortality takes place, it is usually related to immunosuppression or co-infection with other disease agents. For example, sudden death in two captive grey wolves (*Canis lupus*) in apparently good body condition associated with *B. canis* infection could be, according to the authors, secondary to the immunosupression related to captivity, which probably lead to the clinical manifestation [106]. Similarly, marked anemia in a Hokkaido brown bear cub was conceivably caused by the combination of a heavy tick infestation and *Babesia* sp. infection, which was aggravated by stress factors [79]. Another fatal acute infection by *Babesia* sp. was recorded in a captive juvenile African wild dog (*Lycaon pictus*), and was associated with vaccination-induced reduction in its immune competence [35]. In the case of *B. microti*-like group infection, a clinical case with hemolytic anemia and weakness was reported in a free-living juvenile red fox [64]; these clinical signs are similar to those described in infected dogs with babesiosis [23, 24].

Besides these factors, research has shown that historic host-pathogen relationships may be altered by extreme climatic conditions, which may synchronize the temporal and spatial convergence of multiple infectious agents, triggering epidemics with far greater mortality than that produced by a single pathogen. For example, in 1994, epidemics with high mortality in Serengeti lions (*Panthera leo*) were originally attributed to canine distemper virus (CDV) [107], but retrospective analysis revealed that the distemper epidemic coincided with an unusually high prevalence of *Babesia* sp. infection [93]. This was the result of extreme drought conditions with widespread herbivore die-off [108], which according to Munson et al. [93], increased the lion’s exposure to tick-infested starving prey. The combination of high frequency of exposure to ticks and CDV-related immunosuppression caused the hemoparasite infections to become fulminate [93, 109]. Another episode of mortality in 2001 due to CDV that struck the nearby Ngorongoro Crater lion population was also associated with an unusually high prevalence of *Babesia* sp. infection [93].

#### *Rangelia* spp.

Clinical signs have been reported in wild foxes naturally infected with *R. vitalii*. In one case, a wild female pampas fox was found with physical debilitation, motor incordination, dehydratation, pale mucous membranes, apathy, and hypothermia [72]. In another two cases, no signs associated to typical clinical rangeliosis were detected. These included a pampas fox that was in good body condition, with moderately pale mucosae, and a crab-eating fox showing myoclonic rear limbs, paresthesia of front limbs and distinctly pale conjuctivae and oral mucosae [73]. In both canids, necropsy revealed generalized jaundice and histopathology examination showed *R. vitalii* in endothelial cells of liver, stomach, heart, kidney, lungs, lymph nodes, and gall bladder [73]. The significance of rangeliosis at the population level has not been investigated.

#### *Cytauxzoon* spp.

Wild felids naturally infected with *Cytauxzoon* spp. rarely display clinical signs. Among free-living felids, there is only one report of a naturally-infected young bobcat with acute cytauxzoonosis. This animal suffered from severe anemia and irregular respiration [110]. In fact, bobcats rarely display clinical illness, and when disease occurs, it is usually from mild to moderate, and schizogenous replication is limited [85]. Parasitized Iberian lynx were always apparently healthy [75, 77]. Brazilian wild felids did not appear to have clinical signs either [111]. Among captive felids, the death of a seven-year-old tiger (*Panthera tigris*) in a Florida Zoo from acute fever and cellular necrosis after a two-day history of anorexia and lethargy, was reported [112]. Cytauxzoonosis was diagnosed by histological changes including large numbers of intravascular macrophages containing developmental stages of *Cytauxzoon* sp. in the lungs, spleen, liver and bone marrow. The origin of the infected ticks was undetermined [112]. In another case, a captive male cougar (*Puma concolor*) infected with *C. felis* showed anorexia, depression, lethargy and anemia, but not fever, and was ultimately euthanized because of a condition attributed to diabetes mellitus; in this case, *Cytauxzoon* infection was diagnosed by PCR [113]. Fatal cytauxzoonosis was also reported in another tiger born and kept in a German Zoo presenting with anorexia, lethargy and dyspnea [114], and in a 6-month-old captive-reared lion (*Panthera leo*) cub and its mother living in the same exhibit in Brazil [88].

Finally, the above-mentioned case described by Jinnai et al. [79] of an anemic Japanese brown bear cub separated from his mother soon after emerging from hibernation is noteworthy. The cub was heavily infested with ticks and was found to be co-infected by *Cytauxzoon* sp. (showing 90.1 % and 90.2 % identities with *C. felis* and *C. manul*, respectively) and *Babesia* sp. The stress derivated from being lost and the intense tick infestation probably led to the development of clinical illness. Moreover, according to Jinnai et al. [79], the presence of multiple genotypes can result in recombination, bringing benefits for the parasite such as genetic modifications in virulence, transmission, induction of immunity and drug resistance.

### Role of wildlife in the epidemiology of piroplasmids

As shown in the present review, there is abundant evidence of piroplasmid infections in wild carnivores worldwide, in some circumstances displaying high prevalences. There are species of abundant wild carnivores that could serve as reservoirs for piroplasmids, and a wide range of potential vectors that may allow these parasites to maintain endemic sylvatic life-cycles in their geographical distribution area. This could potentially lead to the transmission of infection to domestic carnivores, especially in peri-urban and urban environments [8, 60, 90]. In this regard, many wild reservoir hosts (e.g. red fox, golden jackal and raccoon) are increasing in number and expanding their geographical ranges, thus increasing intra- and interspecies contact risk with domestic carnivores [115]. However, a high prevalence of infection alone does not demonstrate that the species in question acts as a reservoir. In addition, many species of wild carnivores are not abundant, and probably unable to maintain a pathogen in the absence of dogs or another reservoir.

As already outlined, there is some consensus about the bobcat as the natural reservoir of *C. felis* in North America [85]. Infections with *C. felis* in domestic cats in enzootic areas occur when the cats become incorporated into the naturally occurring cycle between bobcats [86, 116] and the tick vector [105]. Cats living close to wooded areas or less intensely managed land are more likely to become infected [105]. Pumas may be an additional natural reservoir for *C. felis* in the United States [117–119]. Brazilian wild felids may be a potential reservoir for *Cytauxzoon* sp. because, as mentioned previously, they did not appear to be clinically infected [111]. Regarding the Iberian lynx, its role as a reservoir is doubtful due to its extremely low population size (less than 300 individuals). Moreover, the only domestic cat diagnosed with *Cytauxzoo*n infection with no clinical data available in Spain was located far from lynx distribution areas [120]. Most likely, the natural reservoir in Iberia is the wildcat (*Felis silvestris silvestris*), which is more abundant, has a broader distribution area and frequently interacts with domestic cats. Though no data is available in Spanish wildcats, a recent study reported that 19 % of Italian wildcats were positive for piroplasmid infection and three sequenced amplicons clustered with the Italian, Spanish, French and Romanian *Cytauxzoon* spp. isolates and with *C. manul* [121].

On the other hand, experimental infection of domestic cats with *C. manul* from Pallas's cats was successful, with cats developing a low but noticeable and persitent parasitemia. Thus, potential interspecies transmission is likely [122]. However, the predominance of subclinical erythroparasitemia and the evidence of persistent infection in the only endemic focus described in Europe (Trieste, Italy) in free-ranging domestic cats support the hypothesis that the domestic cat may serve as a reservoir host for this infection [123].

On the other hand, a growing body of evidence (Table 1) suggest that other wild carnivore species may serve as reservoirs of pirolasmids. For example, the high prevalence of *B. microti*-like group infection in red foxes in diverse locations suggests that this species may be the natural host and sylvatic reservoir of the parasite [49, 54]. Similarly, the raccoon may be the natural host of two or more species of *Babesia* (see above). Both wild foxes and racoons often have peridomestic habits that may facilitate inter-species transmission with dogs. Finally, it has been proposed that crab-eating fox could act as natural reservoir of *R. vitalii* in rural and periurban areas in Brazil [73].

Few attempts have been made to demonstrate susceptibility in a species of wild carnivore experimentally [28, 122, 124]. In one study, coyotes (*Canis latrans*) experimentally infected with *B. gibsoni* developed a maximum parasitemia of 8–11 % infected red blood cells, but this did not significantly affect the health of the coyotes. The long duration of the infection, the high level of parasitemia and the absence of clinical disease suggested that coyotes could serve as potential reservoirs [28].

### Zoonotic implications

Zoonotic species are found among *Babesia* species, but humans are not natural hosts of *Theileria* spp. or *Cytauxzoon* spp. Humans can, however, be accidental hosts for numerous *Babesia* spp. [3, 5]. Yet, as far as it is known, none of the piroplasmids infecting wild carnivores are zoonotic. Nevertheless, Hersh et al. [82] described the presence of the zoonotic *B. microti* in *I. scapularis* ticks retrieved from raccoons and skunks in the USA. If these ticks were infected after biting these carnivore hosts, this would have major implications for *B. microti* dynamics. Therefore, raccoons and skunks could play a critical role in the transmission of the disease in the USA as mechanical dispersers of infected ticks. Their role would depend on their *B. microti*-infected tick loads and relative tick abundance [82]. Nevertheless, infections of carnivores by *B. microti* have never been confirmed, and references to *B. microti* infections in carnivores may represent *B. microti*-like infections (see above).

### Potential impact on wild carnivore conservation

Diseases can have a profound effect on wildlife populations. In fact, one of the most repeated examples of the impact of a pathogen in a wild carnivore population was the canine distemper epidemic in Serengeti lions [107]. However, as mentioned above, subsequent analyses showed that levels of *Babesia* in lions were significantly higher during the 1994 and 2001 epidemics, and that CDV probably acted as an immunosuppressive agent that caused babesiosis to fulminate [93, 109]. This is the only available evidence of a piroplasmid having a demonstrable negative effect on the population dynamics of a wild carnivore. However, evidence of piroplasmid-related disease has been reported in some individuals (see above, and Tables 1 and 2).

On the other hand, wild carnivores are sometimes captured for translocation to establish new populations or reinforce existing ones. Alternatively, in the context of *ex situ* conservation actions, captive-bred animals are released into the wild [8, 67]. All of these management actions can create favorable conditions for the development of clinical piroplasmosis in the animals. Stress-mediated recrudescence of latent infections can also take place. For example, a case of mortality caused by *Theileria* sp. in a wild ungulate after a translocation was attributed to stress factors resulting from the translocation [95]. On the other hand, released individuals might fortuitously introduce new species or strains of a parasite into a naïve population. For example, during Iberian lynx conservation efforts, lynx from the northern population (where *Cytauxzoon* sp. is present) were translocated to the southern one (Doñana, where the parasite has never been detected). This may eventually pose a risk if the southern population lacks acquired immunity against the parasite.

### Knowledge gaps and future research perspectives

To better understand the role of wild carnivores in the epidemiology of piroplasmoses and to determine eventual conservation threats for endangered carnivores, it is imperative that research be conducted to fill the gaps existing in the knowledge of the natural history of the different species of piroplasmids. These gaps may include:

- The exact determination and classification of the causative agent for diverse piroplasmid infections in wildlife.
- The identity of the vector/s and/or reservoir/s for many agents (e.g. *Cytauxzoon* sp. in Europe, Asia, and South America, and for "*B. microti*-like" or *R. vitalii*). These data are extremely important to understand the disease dynamics of piroplasmoses and to determine potential distribution areas of the disease.
- The investigation of the critical role of ticks in the dynamics of piroplasmoses. For some piroplasmids, such as *Cytauxzoon* spp. in Eurasia, the competent vector is still unknown. It is also necessary to determine the ability of ticks to serve as reservoirs in the absence of the vertebrate host, and the duration of infectivity in the tick vector [3].
- The confirmation of the competence of suspected wild reservoirs to infect the tick vector through xenodiagnosis.
- The investigation of alternative ways of piroplasmid transmission (transplancental, direct) and its role in the maintenance of piroplasmids in the wild in the absence of a tick vector.
- Improved economical and sensitive serological tests for use in the cases where parasites may be difficult to detect by direct methods, and epidemiological surveys in wild populations.
- Improvement of the available molecular biology tools for characterization of piroplasmids infecting wild carnivores, and for comparison with domestic animal-derived sequences.

## Conclusions

Piroplasmid infection is a common feature of wild carnivores wherever it has been investigated, but conversely, there is little information about its role in the epidemiology of the disease. Wild carnivores belong to the same Order as dogs and cats, sharing several disease agents. Some species, such as the red fox, are widespread and in some cases can have high local population abundances. In addition, some wild carnivores often live in sympatry with high-density human and domestic carnivore populations, facilitating inter-species transmission. For example, foxes infected with *B. microti*-like group were frequently detected in the Barcelona metropolitan area [83]. Moreover, outdoor activities such as hiking are increasingly popular, providing an opportunity for ticks to infest domestic dogs accompanying people in natural environments [125]. In conclusion, the research focusing on piroplasmoses in wild carnivores remains in its early stages and many research opportunities exist.

## Abbreviations

CDV: Canine distemper virus
